# Hospitalization Rates and Characteristics of Children Aged <18 Years Hospitalized with Laboratory-Confirmed COVID-19 — COVID-NET, 14 States, March 1–July 25, 2020

**DOI:** 10.15585/mmwr.mm6932e3

**Published:** 2020-08-14

**Authors:** Lindsay Kim, Michael Whitaker, Alissa O’Halloran, Anita Kambhampati, Shua J. Chai, Arthur Reingold, Isaac Armistead, Breanna Kawasaki, James Meek, Kimberly Yousey-Hindes, Evan J. Anderson, Kyle P. Openo, Andy Weigel, Patricia Ryan, Maya L. Monroe, Kimberly Fox, Sue Kim, Ruth Lynfield, Erica Bye, Sarah Shrum Davis, Chad Smelser, Grant Barney, Nancy L. Spina, Nancy M. Bennett, Christina B. Felsen, Laurie M. Billing, Jessica Shiltz, Melissa Sutton, Nicole West, H. Keipp Talbot, William Schaffner, Ilene Risk, Andrea Price, Lynnette Brammer, Alicia M. Fry, Aron J. Hall, Gayle E. Langley, Shikha Garg, COVID-NET Surveillance Team, COVID-NET Surveillance Team, Ashley Coates, Pam Daily Kirley, Tanya Libby, Jeremy Roland, Nisha Alden, Rachel Herlihy, Sarah McLafferty, Paula Clogher, Hazal Kayalioglu, Amber Maslar, Adam Misiorski, Linda Niccolai, Danyel Olson, Christina Parisi, Emily Fawcett, Siyeh Gretzinger, Katelyn Lengacher, Jeremiah Williams, David Blythe, Alicia Brooks, Rachel Park, Michelle Wilson, Kathryn Como-Sabetti, Richard Danila, Cory Cline, Kathy Angeles, Nancy Eisenberg, Kristina Flores, Caroline Habrun, Emily Hancock, Sarah Khanlian, Meaghan Novi, Erin Phipps, Yadira Salazar-Sanchez, Elizabeth Dufort, Alison Muse, Sophrena Bushey, Maria Gaitan, RaeAnne Kurtz, Ama Owusu-Dommey, Lindsey Snyder, Katherine Michaelis, Kylie Seeley, Tiffanie Markus, Ryan Chatelain, Andrea George, Mary Hill, Laine McCullough, Melanie Spencer, Ashley Swain, Keegan McCaffrey, Rachel Holstein, Seth Meador, Jonathan Wortham

**Affiliations:** ^1^CDC COVID-NET Team; ^2^US Public Health Service, Rockville, Maryland; ^3^Eagle Global Scientific, Atlanta, Georgia; ^4^Cherokee Nation Assurance, Arlington, Virginia; ^5^California Emerging Infections Program, Oakland, California; ^6^School of Public Health, University of California, Berkeley, Berkeley, California; ^7^University of Colorado Anschutz Medical Campus, Aurora, Colorado; ^8^Colorado Department of Public Health & Environment, Denver, Colorado; ^9^Connecticut Emerging Infections Program, Yale School of Public Health, New Haven, Connecticut; ^10^Departments of Pediatrics and Medicine, Emory University School of Medicine, Atlanta, Georgia; ^11^Emerging Infections Program, Atlanta Veterans Affairs Medical Center, Atlanta, Georgia; ^12^Iowa Department of Public Health, Des Moines, Iowa; ^13^Maryland Department of Health, Baltimore, Maryland; ^14^Michigan Department of Health and Human Services, Lansing, Michigan; ^15^Minnesota Department of Health, St. Paul, Minnesota; ^16^New Mexico Emerging Infections Program, Albuquerque, New Mexico; ^17^New Mexico Department of Health, Santa Fe, New Mexico; ^18^New York State Department of Health, Albany, New York; ^19^University of Rochester School of Medicine and Dentistry, Rochester, New York; ^20^Ohio Department of Health, Columbus, Ohio; ^21^Public Health Division, Oregon Health Authority, Portland, Oregon; ^22^Vanderbilt University Medical Center, Nashville, Tennessee; ^23^Salt Lake County Health Department, Salt Lake City, Utah.; California Emerging Infections Program; Colorado Department of Public Health & Environment; Connecticut Emerging Infections Program; Yale School of Public Health; Emerging Infections Program; Georgia Department of Health; Veterans Affairs Medical Center; Foundation for Atlanta Veterans Education and Research; Maryland Department of Health; Maryland Emerging Infections Program,; The Johns Hopkins Bloomberg School of Public Health; Minnesota Department of Health; New Mexico Department of Health; New Mexico Emerging Infections Program; New York State Department of Health; University of Rochester School of Medicine and Dentistry; Oregon Health Authority; Oregon Health &; Science University; Vanderbilt University Medical Center; Salt Lake County Health Department; Utah Department of Health; Oak Ridge Institute for Science and Education and CDC COVID-NET Team; CDC COVID-NET Team.

Most reported cases of coronavirus disease 2019 (COVID-19) in children aged <18 years appear to be asymptomatic or mild (*1*). Less is known about severe COVID-19 illness requiring hospitalization in children. During March 1–July 25, 2020, 576 pediatric COVID-19 cases were reported to the COVID-19–Associated Hospitalization Surveillance Network (COVID-NET), a population-based surveillance system that collects data on laboratory-confirmed COVID-19–associated hospitalizations in 14 states (*2*,*3*). Based on these data, the cumulative COVID-19-associated hospitalization rate among children aged <18 years during March 1–July 25, 2020, was 8.0 per 100,000 population, with the highest rate among children aged <2 years (24.8). During March 21–July 25, weekly hospitalization rates steadily increased among children (from 0.1 to 0.4 per 100,000, with a weekly high of 0.7 per 100,000). Overall, Hispanic or Latino (Hispanic) and non-Hispanic black (black) children had higher cumulative rates of COVID-19–associated hospitalizations (16.4 and 10.5 per 100,000, respectively) than did non-Hispanic white (white) children (2.1). Among 208 (36.1%) hospitalized children with complete medical chart reviews, 69 (33.2%) were admitted to an intensive care unit (ICU); 12 of 207 (5.8%) required invasive mechanical ventilation, and one patient died during hospitalization. Although the cumulative rate of pediatric COVID-19–associated hospitalization remains low (8.0 per 100,000 population) compared with that among adults (164.5),* weekly rates increased during the surveillance period, and one in three hospitalized children were admitted to the ICU, similar to the proportion among adults. Continued tracking of SARS-CoV-2 infections among children is important to characterize morbidity and mortality. Reinforcement of prevention efforts is essential in congregate settings that serve children, including childcare centers and schools.

COVID-NET conducts population-based surveillance for laboratory-confirmed COVID-19–associated hospitalizations in 99 counties^†^ in 14 states (California, Connecticut, Colorado, Georgia, Iowa, Maryland, Michigan, Minnesota, New Mexico, New York, Ohio, Oregon, Tennessee, and Utah), representing all 10 U.S. Department of Health and Human Services regions (*2*,*3*). Laboratory-confirmed COVID-19–associated hospitalizations among residents in a predefined surveillance catchment area who had a positive SARS-CoV-2 molecular test during hospitalization or up to 14 days before admission are included in surveillance. SARS-CoV-2 tests are ordered at the discretion of the treating health care provider. Trained surveillance officers perform medical chart abstractions for all identified cases. Patients aged <18 years hospitalized with COVID-19 during March 1–July 25, 2020, were included in this analysis. Weekly and cumulative COVID-19–associated hospitalization rates were calculated using the number of catchment area residents hospitalized with COVID-19 as the numerator and the National Center for Health Statistics vintage 2019 bridged-race postcensal population estimates as the denominator.^§^ Descriptive analyses were conducted using all available data; however, for clinical interventions, treatments, and outcomes, only those hospitalizations with complete medical chart review and a discharge disposition (i.e., discharged alive or died during hospitalization) were included. Obesity was defined as body mass index (kg/m^2^) ≥95th percentile for age and sex based on CDC growth charts among children aged ≥2 years; this was not evaluated for children <2 years. All analyses were conducted using SAS statistical software (version 9.4; SAS Institute). COVID-NET activities were determined by CDC to be public health surveillance.^¶^ Participating sites obtained approval for COVID-NET surveillance from their respective state and local Institutional Review Boards, as required.

During March 1–July 25, 576 children hospitalized with COVID-19 were reported to COVID-NET. Infants aged <3 months accounted for 18.8% of all children hospitalized with COVID-19 (Table). The median patient age was 8 years (interquartile range [IQR] = 9 months–15 years), and 292 (50.7%) were males. Among 526 (91.3%) children for whom race and ethnicity information were reported, 241 (45.8%) were Hispanic, 156 (29.7%) were black, 74 (14.1%) were white; 24 (4.6%) were non-Hispanic Asian or Pacific Islander; and four (0.8%) were non-Hispanic American Indian/Alaska Native.

**TABLE:** Demographic and clinical characteristics of children aged <18 years hospitalized with COVID-19 — COVID-NET, 14 States,* March 1–July 25, 2020^†^

Characteristic	No./Total no. (%)			
	All ages	0–2 yrs	2–4 yrs	5–17 yrs
**Age (N = 576)**				
0–2 mos	108/576 (18.8)	—	—	—
3–5 mos	20/576 (3.5)	—	—	—
6–11 mos	29/576 (5.0)	—	—	—
12–23 mos	31/576 (5.4)	—	—	—
2–4 yrs	50/576 (8.7)	—	—	—
5–11 yrs	97/576 (16.8)	—	—	—
12–17 yrs	241/576 (41.8)	—	—	—
**Age (N = 576) median (IQR)**	8 yrs (9 mos–15 yrs)			
**Sex (N = 576)**				
Male	292/576 (50.7)	106/188 (56.4)	25/50 (50.0)	161/338 (47.6)
Female	284/576 (49.3)	82/188 (43.6)	25/50 (50.0)	177/338 (52.4)
**Race/Ethnicity (N = 526)**				
NH White	74/526 (14.1)	29/162 (17.9)	5/46 (10.9)	40/318 (12.6)
NH Black	156/526 (29.7)	38/162 (23.5)	17/46 (37.0)	101/318 (31.8)
Hispanic or Latino	241/526 (45.8)	73/162 (45.1)	18/46 (39.1)	150/318 (47.2)
NH American Indian/Alaska Native	4/526 (0.8)	0/162 (—)	0/46 (—)	4/318 (1.3)
NH Asian or Pacific Islander	24/526 (4.6)	13/162 (8.0)	3/46 (6.5)	8/318 (2.5)
Multiple races	3/526 (0.6)	0/162 (—)	1/46 (2.2)	2/318 (0.6)
Unknown	24/526 (4.6)	9/162 (5.6)	2/46 (4.3)	13/318 (4.1)
**Any underlying condition (N = 222)**	94/222 (42.3)	14/65 (21.5)	9/24 (37.5)	71/133 (53.4)
Obesity^§^	42/111 (37.8)	N/A	6/18 (33.3)	36/93 (38.7)
Chronic lung disease	40/222 (18.0)	2/65 (3.1)	4/24 (16.7)	34/133 (25.6)
Asthma	30/222 (13.5)	1/65 (1.5)	0/24 (0)	29/133 (21.8)
Prematurity (gestational age <37 weeks)^¶^	10/65 (15.4)	10/65 (15.4)	N/A	N/A
Neurologic disorder	31/222 (14.0)	6/65 (9.2)	7/24 (29.2)	18/133 (13.5)
Immunocompromised condition	12/222 (5.4)	0/65 (—)	2/24 (8.3)	10/133 (7.5)
Feeding tube dependent	12/222 (5.4)	4/65 (6.2)	3/24 (12.5)	5/133 (3.8)
Chronic metabolic disease	10/222 (4.5)	1/65 (1.5)	0/24 (—)	9/133 (6.8)
Diabetes mellitus	6/222 (2.7)	0/65 (—)	0/24 (—)	6/133 (4.5)
Blood disorders	8/222 (3.6)	0/65 (—)	0/24 (—)	8/133 (6.0)
Sickle cell disease	5/222 (2.3)	0/65 (—)	0/24 (—)	5/133 (3.8)
Cardiovascular disease	7/222 (3.2)	2/65 (3.1)	2/24 (8.3)	3/133 (2.3)
Congenital heart disease	4/222 (1.8)	2/65 (3.1)	1/24 (4.2)	1/133 (0.8)
**Any underlying condition by race/ethnicity (N = 94)**				
NH White	14/94 (14.9)	4/14 (28.6)	0/9 (—)	10/71 (14.1)
NH Black	28/94 (29.8)	3/14 (21.4)	2/9 (22.2)	23/71 (32.4)
Hispanic or Latino	43/94 (45.7)	7/14 (50)	6/9 (66.7)	30/71 (42.3)
NH American Indian/Alaska Native	2/94 (2.1)	0/14 (—)	0/9 (—)	2/71 (2.8)
NH Asian or Pacific Islander	3/94 (3.2)	0/14 (—)	0/9 (—)	3/71 (4.2)
Multiracial	1/94 (1.1)	0/14 (—)	1/9 (11.1)	0/71 (—)
Unknown	3/94 (3.2)	0/14 (—)	0/9 (—)	3/71 (4.2)
**Signs and symptoms (N = 224)**				
Fever/chills	121/224 (54.0)	50/67 (74.6)	13/24 (54.2)	58/133 (43.6)
Inability to eat/poor feeding^¶^	22/67 (32.8)	22/67 (32.8)	N/A	N/A
Nausea/vomiting	69/224 (30.8)	14/67 (20.9)	6/24 (25.0)	49/133 (36.8)
Cough	66/224 (29.5)	17/67 (25.4)	3/24 (12.5)	46/133 (34.6)
Nasal congestion/rhinorrhea	53/224 (23.7)	22/67 (32.8)	5/24 (20.8)	26/133 (19.5)
Shortness of breath/respiratory distress	50/224 (22.3)	9/67 (13.4)	2/24 (8.3)	39/133 (29.3)
Abdominal pain	42/224 (18.8)	2/67 (3.0)	3/24 (12.5)	37/133 (27.8)
Diarrhea	27/224 (12.1)	5/67 (7.5)	3/24 (12.5)	19/133 (14.3)
**Hospitalization length of stay (N = 208) median days (IQR)**	2.5 (1—5)	2 (1—2)	3 (1—4)	3 (2—6)
**Chest radiograph findings (N = 67)**				
Infiltrate/consolidation	44/67 (65.7)	8/15 (53.3)	3/9 (33.3)	33/43 (76.7)
Bronchopneumonia/pneumonia	14/67 (20.9)	2/15 (13.3)	0/9 (—)	12/43 (27.9)
				
Pleural effusion	4/67 (6.0)	0/15 (—)	1/9 (11.1)	3/43 (7.0)
**Chest CT findings (N = 14)**				
Ground glass opacities	10/14 (71.4)	1/1 (100.0)	1/1 (100.0)	8/12 (66.7)
Infiltrate/consolidation	7/14 (50.0)	0/1 (—)	0/1 (—)	7/12 (58.3)
Bronchopneumonia/pneumonia	4/14 (28.6)	0/1 (—)	0/1 (—)	4/12 (33.3)
Pleural effusion	3/14 (21.4)	0/1 (—)	0/1 (—)	3/12 (25.0)
COVID-19 investigational treatment (N = 208)**				
Received treatment	12/208 (5.8)	0/61 (—)	0/24 (—)	12/123 (9.8)
Remdesivir	9/208 (4.3)	0/61 (—)	0/24 (—)	9/123 (7.3)
Azithromycin^††^	6/208 (2.9)	0/61 (—)	0/24 (—)	6/123 (4.9)
Hydroxychloroquine	4/208 (1.9)	0/61 (—)	0/24 (—)	4/123 (3.3)
Convalescent plasma	1/208 (0.5)	0/61 (—)	0/24 (—)	1/123 (0.8)
Lopinavir-ritonavir^§§^	1/208 (0.5)	0/61 (—)	0/24 (—)	1/123 (0.8)
**ICU admission (N = 208)**	69/208 (33.2)	19/61 (31.1)	9/24 (37.5)	41/123 (33.3)
ICU length of stay median days (IQR)	2 (1—5)	1 (1—3)	2 (2—5)	3.5 (1—7)
**Interventions (N = 208)** ^¶¶^				
Invasive mechanical ventilation***	12/207 (5.8)	0/61 (—)	4/24 (16.7)	8/122 (6.6)
BIPAP/CPAP***	8/207 (3.9)	2/61 (3.3)	2/24 (8.3)	4/122 (3.3)
High flow nasal cannula***	5/207 (2.4)	1/61 (1.6)	1/24 (4.2)	3/122 (2.5)
Systemic steroids	19/208 (9.1)	1/61 (1.6)	4/24(16.7)	14/123 (11.4)
IVIG	14/208 (6.7)	1/61 (1.6)	5/24 (20.8)	8/123 (6.5)
Vasopressor	10/208 (4.8)	0/61 (—)	0/24 (—)	10/123 (8.1)
**New clinical discharge diagnoses (N = 208)**				
Pneumonia	23/208 (11.1)	2/61 (3.3)	2/24 (8.3)	19/123 (15.4)
Multisystem inflammatory syndrome in children (MIS-C)^†††^	9/83 (10.8)	1/15 (6.7)	5/15 (33.3)	3/53 (5.7)
Acute respiratory failure	10/208 (4.8)	0/61 (—)	3/24 (12.5)	7/123 (5.7)
Acute kidney injury	6/208 (2.9)	0/61 (—)	0/24 (—)	6/123 (4.9)
Diabetic ketoacidosis	6/208 (2.9)	0/61 (—)	0/24 (—)	6/123 (4.9)
Acute respiratory distress syndrome	4/208 (1.9)	1/61 (1.6)	0/24 (—)	3/123 (2.4)
**Died during hospitalization (N = 208)**	1/208 (0.5)	0/61 (—)	0/24 (—)	1/123 (0.8)

**Abbreviations:** BIPAP = bilevel positive airway pressure; CT = computed tomography; CPAP = continuous positive airway pressure; COVID-19 = coronavirus disease 2019; COVID-NET = COVID-19–Associated Hospitalization Surveillance Network; ICU = intensive care unit; IQR = interquartile range; IVIG = intravenous immune globulin; N/A = not applicable; NH = non-Hispanic.

* California, Connecticut, Colorado, Georgia, Iowa, Maryland, Michigan, Minnesota, New Mexico, New York, Ohio, Oregon, Tennessee, and Utah.

**^†^** Analyses were conducted on all available data; however, for hospitalization length of stay, radiology findings, treatments, ICU admission, interventions, new clinical diagnoses, and outcome, only cases with a complete medical chart review and a discharge disposition (i.e. discharged alive or died during hospitalization) were included.

^§^ Obesity was defined as body mass index (kg/m^2^) ≥95th percentile for age and sex based on CDC growth charts among children aged ≥2 years; this was not evaluated for children <2 years.

^¶^ Data collected only on children aged <2 years.

** Not mutually exclusive treatment categories.

^††^ Given with at least one other COVID-19 investigational treatment.

^§§^ Not given for human immunodeficiency virus infection.

^¶¶^ Two hospitalized children received extracorporeal membrane oxygenation (1 each aged <2 years and 5–17 years). None received renal replacement therapy.

*** Highest level of respiratory support for each case that needed respiratory support.

^†††^ Since June 18, a discharge diagnosis of multisystem inflammatory syndrome in children (MIS-C) was systematically collected through COVID-NET.

The cumulative COVID-19–associated hospitalization rate among children aged <18 years during the surveillance period was 8.0 per 100,000 and was highest among children aged <2 years (24.8); rates were substantially lower in children aged 2–4 years (4.2) and 5–17 years (6.4) (Figure 1). Overall weekly hospitalization rates among children increased steadily during the surveillance period (from 0.1 to 0.4 per 100,000, with a weekly high of 0.7 per 100,000; trend test, p<0.001) (Figure 1). COVID-19–associated hospitalization rates were higher among Hispanic and black children than among white children (Figure 2); the rates among Hispanic and black children were nearly eight times and five times, respectively, the rate in white children.

**FIGURE 1:**
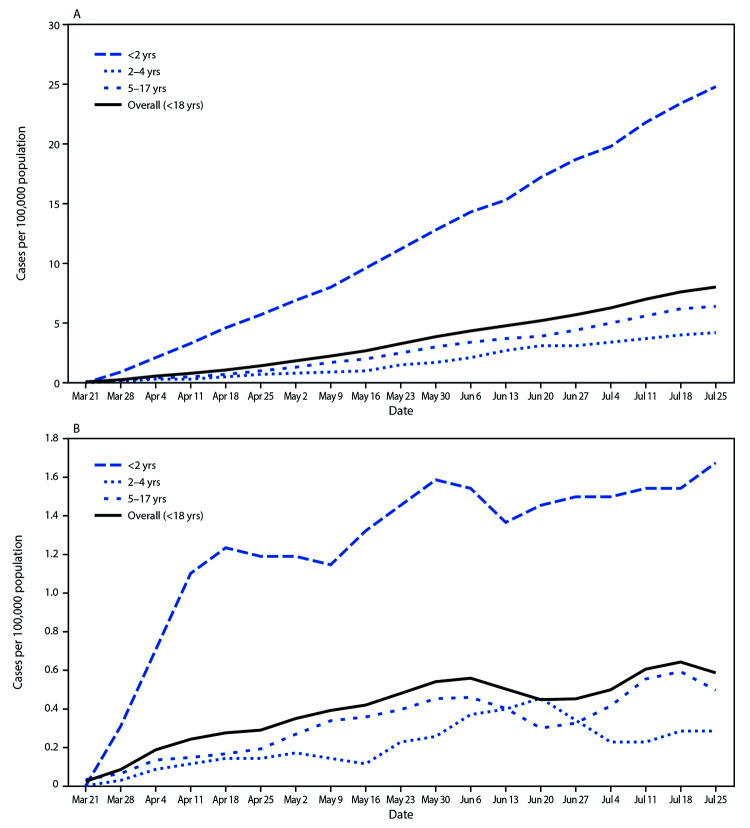
Cumulative (A) and weekly (B) COVID-19–associated hospitalization rates*^,†^ among children aged <18 years, by age group — COVID-NET, 14 states^§^, March 1–July 25, 2020^¶^ **Abbreviation:** COVID-NET = Coronavirus Disease 2019–Associated Hospitalization Surveillance Network. * Number of children in each age group hospitalized with COVID-19 per 100,000 population. ^†^ Figure B shows the 3-week moving average of weekly hospitalization rates for children in each age group hospitalized with COVID-19 per 100,000 population. A trend test was conducted using weighted linear regression, where the weight for each week was the inverse of the variance. Trend test overall (<18 years): p-value <0.001. ^§^ Counties included in COVID-NET surveillance: California (Alameda, Contra Costa, and San Francisco counties); Colorado (Adams, Arapahoe, Denver, Douglas, and Jefferson counties); Connecticut (New Haven and Middlesex counties); Georgia (Clayton, Cobb, DeKalb, Douglas, Fulton, Gwinnett, Newton, and Rockdale counties); Iowa (one county represented); Maryland (Allegany, Anne Arundel, Baltimore, Baltimore City, Calvert, Caroline, Carroll, Cecil, Charles, Dorchester, Frederick, Garrett, Harford, Howard, Kent, Montgomery, Prince George’s, Queen Anne’s, St. Mary’s, Somerset, Talbot, Washington, Wicomico, and Worcester counties); Michigan (Clinton, Eaton, Genesee, Ingham, and Washtenaw counties); Minnesota (Anoka, Carver, Dakota, Hennepin, Ramsey, Scott, and Washington counties); New Mexico (Bernalillo, Chaves, Dona Ana, Grant, Luna, San Juan, and Santa Fe counties); New York (Albany, Columbia, Genesee, Greene, Livingston, Monroe, Montgomery, Ontario, Orleans, Rensselaer, Saratoga, Schenectady, Schoharie, Wayne, and Yates counties); Ohio (Delaware, Fairfield, Franklin, Hocking, Licking, Madison, Morrow, Perry, Pickaway, and Union counties); Oregon (Clackamas, Multnomah, and Washington counties); Tennessee (Cheatham, Davidson, Dickson, Robertson, Rutherford, Sumner, Williamson, and Wilson counties); and Utah (Salt Lake County). ^¶^ Data are preliminary, and case counts and rates for recent hospital admissions are subject to lag. As data are received each week, previous case counts and rates are updated accordingly.

**FIGURE 2:**
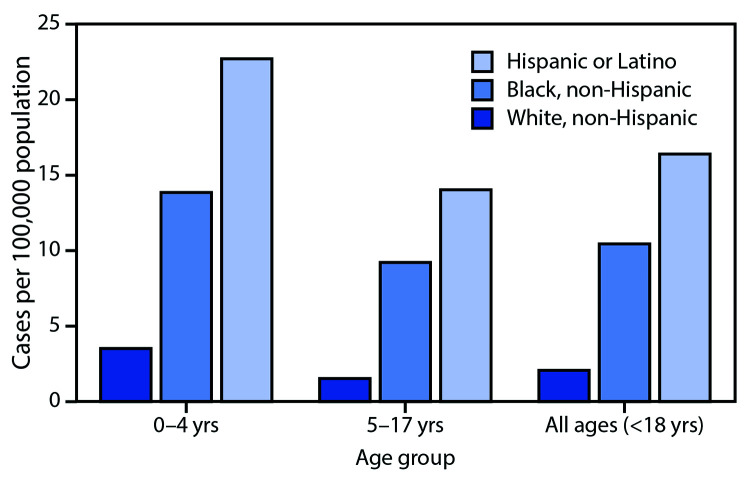
Cumulative COVID-19–associated hospitalization rates* among children aged <18 years, by age group and race/ethnicity — COVID-NET, 14 states^†^, March 1–July 25, 2020^§^^,^^¶^ **Abbreviation:** COVID-NET = Coronavirus Disease 2019–Associated Hospitalization Surveillance Network. * Number of children aged <18 years hospitalized with COVID-19 per 100,000 population. ^†^ Counties included in COVID-NET surveillance: California (Alameda, Contra Costa, and San Francisco counties); Colorado (Adams, Arapahoe, Denver, Douglas, and Jefferson counties); Connecticut (New Haven and Middlesex counties); Georgia (Clayton, Cobb, DeKalb, Douglas, Fulton, Gwinnett, Newton, and Rockdale counties); Iowa (one county represented); Maryland (Allegany, Anne Arundel, Baltimore, Baltimore City, Calvert, Caroline, Carroll, Cecil, Charles, Dorchester, Frederick, Garrett, Harford, Howard, Kent, Montgomery, Prince George’s, Queen Anne’s, St. Mary’s, Somerset, Talbot, Washington, Wicomico, and Worcester counties); Michigan (Clinton, Eaton, Genesee, Ingham, and Washtenaw counties); Minnesota (Anoka, Carver, Dakota, Hennepin, Ramsey, Scott, and Washington counties); New Mexico (Bernalillo, Chaves, Dona Ana, Grant, Luna, San Juan, and Santa Fe counties); New York (Albany, Columbia, Genesee, Greene, Livingston, Monroe, Montgomery, Ontario, Orleans, Rensselaer, Saratoga, Schenectady, Schoharie, Wayne, and Yates counties); Ohio (Delaware, Fairfield, Franklin, Hocking, Licking, Madison, Morrow, Perry, Pickaway, and Union counties); Oregon (Clackamas, Multnomah, and Washington counties); Tennessee (Cheatham, Davidson, Dickson, Robertson, Rutherford, Sumner, Williamson, and Wilson counties); and Utah (Salt Lake County). ^§^ Data are preliminary, and case counts and rates for recent hospital admissions are subject to lag. As data are received each week, prior case counts and rates are updated accordingly. As of July 25, 2020, 50 (8.7%) of 576 pediatric hospitalized cases were missing data on race and ethnicity. ^¶^ Rates are not shown among non-Hispanic Asian or Pacific Islanders and non-Hispanic American Indian/Alaska Natives because of small case counts, leading to unstable estimates. All non-Hispanic American Indian/Alaska Native hospitalized children were aged 5–17 years.

Among 222 (38.5%) of 576 children with information on underlying medical conditions, 94 (42.3%) had one or more underlying conditions (Table). The most prevalent conditions included obesity (37.8%), chronic lung disease (18.0%), and prematurity (gestational age <37 weeks at birth, collected only for children aged <2 years) (15.4%). Hispanic and black children had higher prevalences of underlying conditions (45.7% and 29.8%, respectively) compared with white children (14.9%). Reported signs and symptoms upon hospital admission differed by age: fever or chills were the most common sign and symptom overall (54%) and were most prevalent among children aged <2 years (74.6%). Gastrointestinal symptoms, including nausea or vomiting, abdominal pain, or diarrhea, were reported by 42% of hospitalized children overall.

A medical chart review was completed for 208 (36.1%) children. Median duration of hospitalization was 2.5 days (IQR = 1–5 days). Among 67 children who had a chest radiograph during hospitalization, 44 (65.7%) radiographs showed an infiltrate or consolidation. Among 14 children with chest computed tomography results available, ground-glass opacities (a nonspecific sign indicating infection or alveolar disease) was reported in 10. COVID-19 investigational treatments were only administered to 12 (5.8%) children, all aged 5–17 years; nine received remdesivir. Intravenous immunoglobulin was received by 14 of 208 (6.7%) children. Sixty-nine children (33.2%) were admitted to the ICU for a median of 2 days (IQR = 1–5 days). Invasive mechanical ventilation was required by 12 (5.8%) of 207 children. Since June 18, a discharge diagnosis of multisystem inflammatory syndrome in children (MIS-C) has been systematically collected**; overall, nine (10.8%) of 83 children with completed chart reviews for whom information about MIS-C was systematically collected received a diagnosis of MIS-C. Among 208 children with a discharge disposition, one child (0.5%) with multiple underlying conditions died during hospitalization.

## Discussion

Since March 1, 2020, COVID-NET has identified 576 pediatric COVID-19–associated hospitalizations. Although the cumulative COVID-19–associated hospitalization rate among children is low compared with that among adults, weekly hospitalization rates in children increased during the surveillance period. Children can develop severe COVID-19 illness; during the surveillance period, one in three children were admitted to the ICU. Hispanic and black children had the highest rates of COVID-19–associated hospitalization.

Continued surveillance will allow for further characterization of the burden and outcomes of COVID-19–associated hospitalizations among children. These data will help to better define the clinical spectrum of disease in children and the contributions of race and ethnicity and underlying medical conditions to hospitalizations and outcomes. 

Reasons for disparities in COVID-19-associated hospitalization rates by race and ethnicity are not fully understood. This report found the highest rates of COVID-19-associated hospitalization among Hispanic children. Similarly, a recent study from the Baltimore-District of Columbia region found a higher prevalence of SARS-CoV-2 infection in the Hispanic community compared with that in other racial and ethnic communities (*4*). Although hospitalization rates were lower for Hispanic persons than for black and white persons, hospitalized Hispanic patients were more likely to be younger (aged <44 years) (*4*). It has been hypothesized that Hispanic adults might be at increased risk for SARS-CoV-2 infection because they are overrepresented in frontline (e.g., essential and direct-service) occupations with decreased opportunities for social distancing, which might also affect children living in those households (*4*). During the 2009 influenza A H1N1 pandemic, pediatric mortality rates also were higher among underrepresented ethnic groups in a study from England (*5*).

Forty-two percent of children in this analysis had one or more underlying medical conditions, with higher prevalences among Hispanic and black children. This suggests that the presence of underlying conditions place children at higher risk for COVID-19-associated hospitalizations and that observed disparities might in part be related to the higher prevalence of underlying conditions among hospitalized Hispanic and black children compared with those among white children. This study, along with other studies of hospitalized children with COVID-19, found that obesity was the most prevalent underlying medical condition (*6*,*7*). Childhood obesity affects almost one in five U.S. children and is more prevalent in black and Hispanic children (*8*); therefore, understanding the underlying pathophysiologic association between obesity and SARS-CoV-2 infection is important to identifying possible clinical interventions and preventive strategies to reduce the risk for hospitalization.

This report and others have found that, although one third of children hospitalized with COVID-19 were admitted to the ICU, the case-fatality rate remains low, even among children hospitalized with more severe COVID-19–associated complications, such as MIS-C (*6*,*7*,*9*). By comparison, among U.S. children hospitalized with seasonal influenza virus infection, estimates of ICU admissions have ranged from 16% to 25% among hospitalized children without and with underlying medical conditions, respectively, and reports of in-hospital deaths also are rare (<1%) (*10*). The percentage of ICU admission was similar among children (33.2%) and adults (32.0%) reported to COVID-NET; however, invasive mechanical ventilation was required less frequently in children (5.8%) than in adults (18.6%) (*3*). Continued monitoring of hospitalizations, ICU admissions, and mortality among children is important to understand potential risk factors for severe outcomes.

The findings in this report are subject to at least five limitations. First, laboratory confirmation is dependent on clinician-ordered SARS-CoV-2 molecular testing. Rates likely are underestimates; cases can be missed because of test availability, test performance, and provider or facility testing practices. Second, hospitalization rates by age group and race/ethnicity are preliminary and might change as additional cases are identified during the surveillance period. Third, analysis of interventions, treatments, and outcomes was based on a convenience sample of children with a final disposition and complete chart reviews. A higher proportion of included children were aged <6 months, and two sites contributed more than half of cases; however, compared with other single-center or state-based studies, COVID-NET is more geographically and racially diverse (*2*). Approximately 60% of pediatric hospitalizations reported to COVID-NET have not had a chart review, and this sample might be biased. In the future, COVID-NET plans to have complete, population-based data on hospitalized children. Finally, COVID-NET did not systematically collect information on MIS-C until June 18. In addition, given that molecular tests can miss approximately half of patients with MIS-C despite serologic or epidemiologic evidence of a past SARS-CoV-2 infection (*9*), COVID-NET surveillance likely underestimates the percentage of MIS-C cases among SARS-CoV-2 infections in children.

Using a multisite, geographically diverse network, this report found that children with SARS-CoV-2 infection can have severe illness requiring hospitalization and intensive care. Improved understanding of the social determinants of health is needed to inform and reduce disparities as evidenced by pediatric COVID-19-associated hospitalization rates. Similar to the general population, children should be encouraged to wash their hands often and continue social distancing, and children aged ≥2 years should wear a mask when around persons outside of their families to reduce the risk for SARS-CoV-2 infection and transmission to others. Ongoing monitoring of hospitalization rates, clinical characteristics, ICU admission, and outcomes in the pediatric population is important to further characterize the morbidity and mortality of COVID-19 in children.

**Summary**

**What is already known about this topic?**

Most reported SARS-CoV-2 infections in children aged <18 years are asymptomatic or mild. Less is known about severe COVID-19 in children requiring hospitalization.

**What is added by this report?**

Analysis of pediatric COVID-19 hospitalization data from 14 states found that although the cumulative rate of COVID-19–associated hospitalization among children (8.0 per 100,000 population) is low compared with that in adults (164.5), one in three hospitalized children was admitted to an intensive care unit.

**What are the implications for public health practice?**

Children are at risk for severe COVID-19. Public health authorities and clinicians should continue to track pediatric SARS-CoV-2 infections. Reinforcement of prevention efforts is essential in congregate settings that serve children, including childcare centers and schools.

## Footnotes

* https://www.cdc.gov/coronavirus/2019-ncov/covid-data/covidview/index.html.

† Counties in COVID-NET surveillance: California (Alameda, Contra Costa, and San Francisco counties); Colorado (Adams, Arapahoe, Denver, Douglas, and Jefferson counties); Connecticut (New Haven and Middlesex counties); Georgia (Clayton, Cobb, DeKalb, Douglas, Fulton, Gwinnett, Newton, and Rockdale counties); Iowa (one county represented); Maryland (Allegany, Anne Arundel, Baltimore, Baltimore City, Calvert, Caroline, Carroll, Cecil, Charles, Dorchester, Frederick, Garrett, Harford, Howard, Kent, Montgomery, Prince George’s, Queen Anne’s, St. Mary’s, Somerset, Talbot, Washington, Wicomico, and Worcester counties); Michigan (Clinton, Eaton, Genesee, Ingham, and Washtenaw counties); Minnesota (Anoka, Carver, Dakota, Hennepin, Ramsey, Scott, and Washington counties); New Mexico (Bernalillo, Chaves, Dona Ana, Grant, Luna, San Juan, and Santa Fe counties); New York (Albany, Columbia, Genesee, Greene, Livingston, Monroe, Montgomery, Ontario, Orleans, Rensselaer, Saratoga, Schenectady, Schoharie, Wayne, and Yates counties); Ohio (Delaware, Fairfield, Franklin, Hocking, Licking, Madison, Morrow, Perry, Pickaway, and Union counties); Oregon (Clackamas, Multnomah, and Washington counties); Tennessee (Cheatham, Davidson, Dickson, Robertson, Rutherford, Sumner, Williamson, and Wilson counties); and Utah (Salt Lake County).

§
https://www.cdc.gov/coronavirus/2019-ncov/covid-data/covidview/purpose-methods.html .

¶ US Department of Health and Human Services, Title 45 Code of Federal Regulations 46, Protection of Human Subjects.

** MIS-C is a hyperinflammatory condition that can affect multiple organs in a child who has a current or recent infection with SARS-CoV-2.
